# Supplementary material for: Interdependent action of KH domain proteins Krr1 and Dim2 drive the 40S platform assembly
Source: Nat Commun. 2017 Dec 20;8:2213. doi: 10.1038/s41467-017-02199-4 (PMC5738357; doi:10.1038/s41467-017-02199-4)
Supplement: Supplementary file 1 — Supplementary Information [file 41467_2017_2199_MOESM1_ESM.pdf]

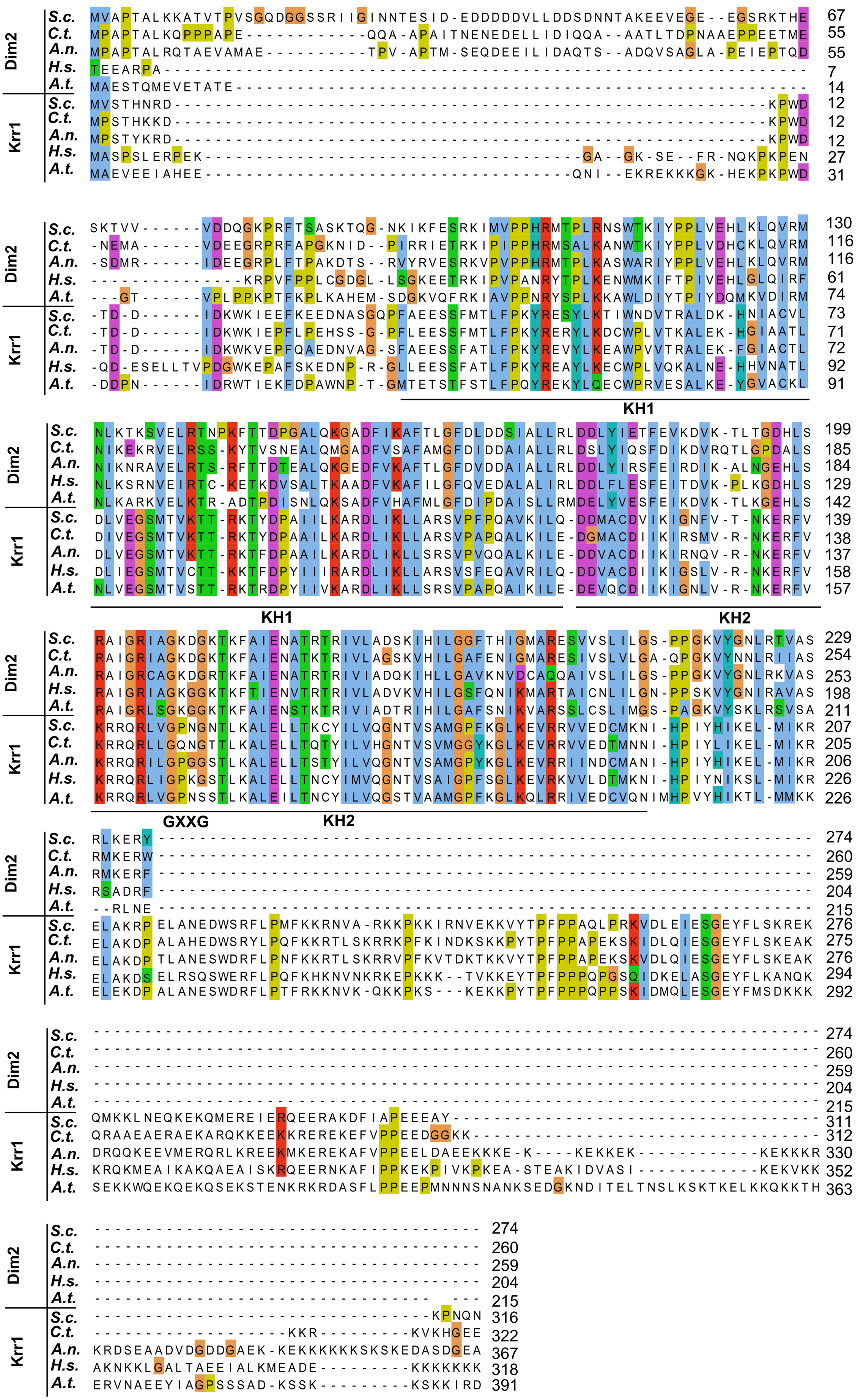
Dim2
Krr1
S.c.
C.t.
A.n.
H.s.
A.t.
KH1
KH2
GXXG

Supplementary Figure 1. Multiple-sequence alignment of KH domain proteins Dim2 and Krr1 from different species. For the alignment, orthologs from the following species were used*: Saccharomyces cerevisiae* (*S.c.*)*, Chaetomium thermophilum* (*C.t.*)*, Aspergillus niger* (*A.n.*)*, Homo sapiens* (*H.s.*) *and Arabidopsis thaliana* (*A.t.*). The default color scheme of ClustalX/Jalview[1] was used with e.g. hydrophobic residues in blue, acidic residues in violet and basic residues in red. Both proteins contain two tandem KH domains (KH1, KH2) in row. In both Dim2 and Krr1, KH1 (KH-like) lacks the conserved GXXG motif, predicted to be required for RNA/DNA binding, which however is present in KH2 of Dim2 and Krr1. Krr1 contains a conserved C-terminal extension absent in Dim2.

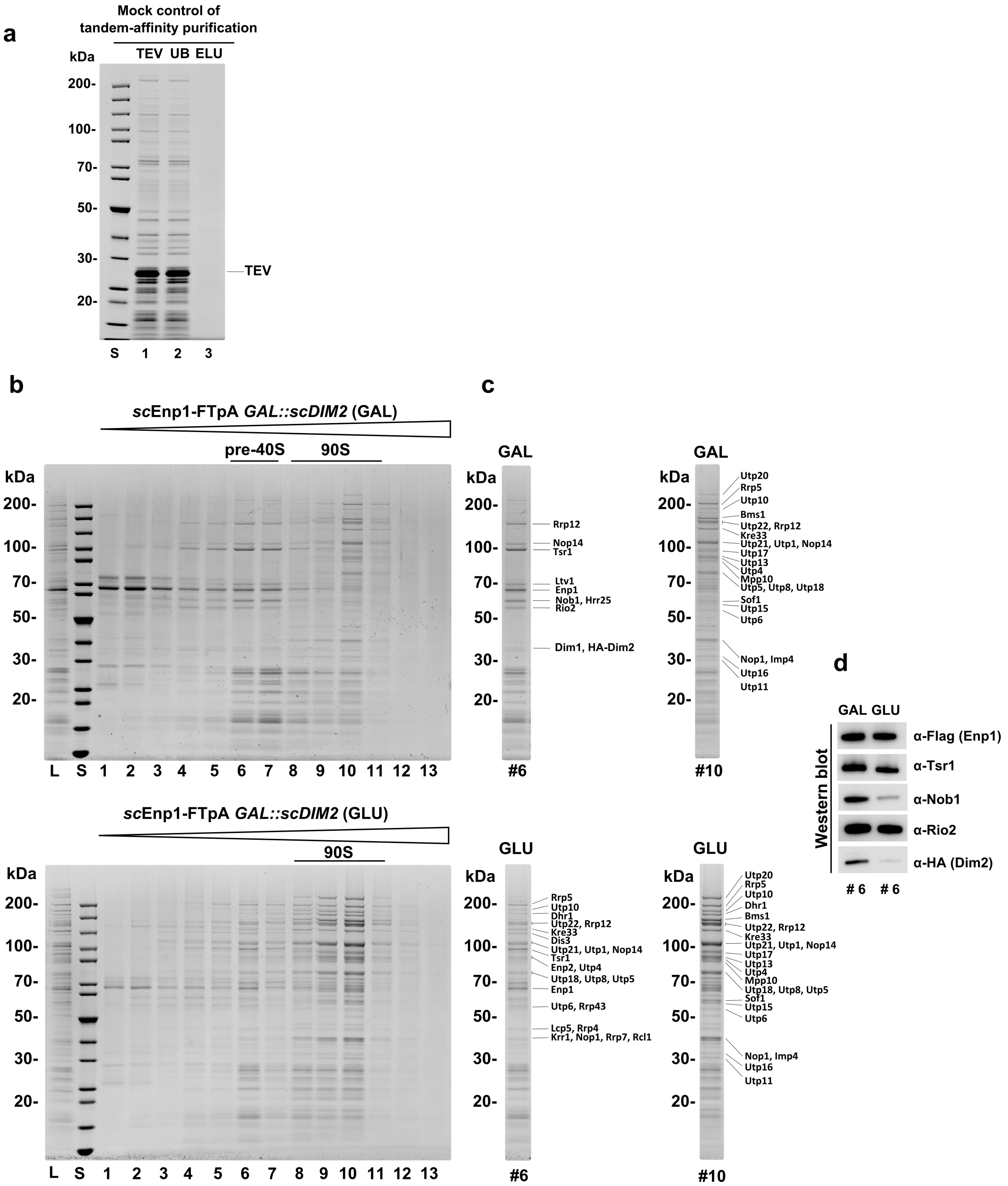

Supplementary Figure 2. Dim2 depletion leads to arrest of 90S biogenesis visualized by sucrose gradient analysis of Enp1-FTpA derived particles. (a) Mock control revealing the specificity of split-tag tandem-affinity purification. Wild-type yeast W303 lysate, devoid of tagged bait proteins, was passed first over IgG-sepharose, followed by TEV elution and a second affinity-purification step via α-FLAG beads. The final FLAG eluate was TCA precipitated and analyzed by SDS-PAGE and Coomassie staining. Loading: 10% of TEV eluate (lane 1), 10% of the unbound fraction (lane 2), 85% of TCA-precipitated Flag eluate (lane 3). S, molecular weight

protein standard. (b) Eluates derived from *sc*Enp1-FTpA tandem affinity-purification under conditions of Dim2 expression (GAL) and depletion (GLU, 8h), respectively, were analyzed by sucrose gradient (15-40%) centrifugation. Gradient fractions (1-13) were TCA-precipitated and analyzed by SDS-PAGE (4-12%) and Coomassie staining. (c) Shown are selected fractions 6 and 10 from the sucrose gradient analysis, for which the labeled bands were identified by mass spectrometry. (d) Western blots analysis of sucrose gradient fractions 6 under Dim2 expression (GAL) and Dim2 repression (GLU) conditions with the indicated antibodies.

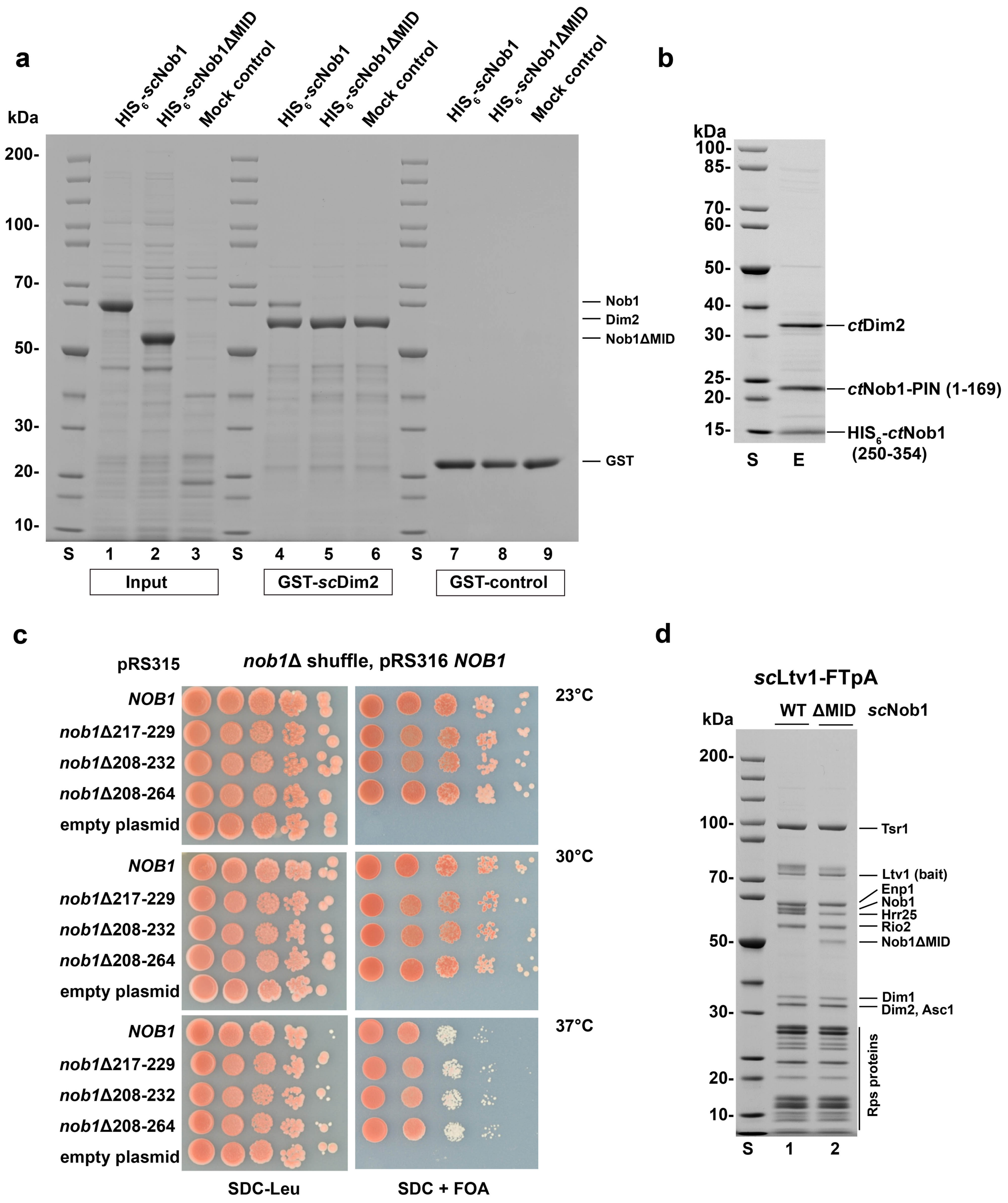

Supplementary Figure 3. Biochemical and *in vivo* analyses of the conserved Dim2-Nob1 interaction. (a) *In vitro* binding assay using yeast *Saccharomyces cerevisiae* GST-*sc*Dim2 and indicated HIS_6-*sc*Nob1 constructs (lanes 1-2). BL21 extract was used as mock control (lane 3). After the binding reaction, final SDS eluates (lanes 4-9) were analyzed by SDS-PAGE and Coomassie staining. Note that full-length Nob1, but not Nob1ΔMID

(Δ 208-264) binds to Dim2. S, molecular weight protein standard. (b) Complex formation between distinct Nob1 domains and Dim2. Recombinant *ct*Dim2, *ct*Nob1 PIN (1-169) and HIS_6-*ct*Nob1 Mid-ZnF (250-354) were co-expressed in *E. coli* Bl21 and affinity-purified utilizing SP-Sepharose and Ni-NTA. The final eluate was analyzed by SDS-PAGE (4-12%) and Coomassie staining. S, molecular weight protein standard; E, eluate. (c) Yeast Nob1 shuffle strain was transformed with single copy plasmids, either empty (pRS315), or containing the indicated *NOB1* constructs, all under the authentic *NOB1* promoter and terminator. Growth was analyzed after three days at the indicated temperatures on SDC-Leu and SDC + FOA plates. (d) Tandem affinity purification of integrated *sc*Ltv1-FTpA from the shuffled yeast Nob1 shuffle strain, carrying either plasmid based *sc*Nob1 WT or *sc*Nob1 ΔMID (Δ208-264). The two consecutive purification steps involve the ProtA- in the first and the Flag-tag in the second step. Final Flag eluates were TCA-precipitated and analyzed by SDS-PAGE (4-12%) and Coomassie staining. The indicated protein bands were identified by mass spectrometry. S, molecular weight protein standard.

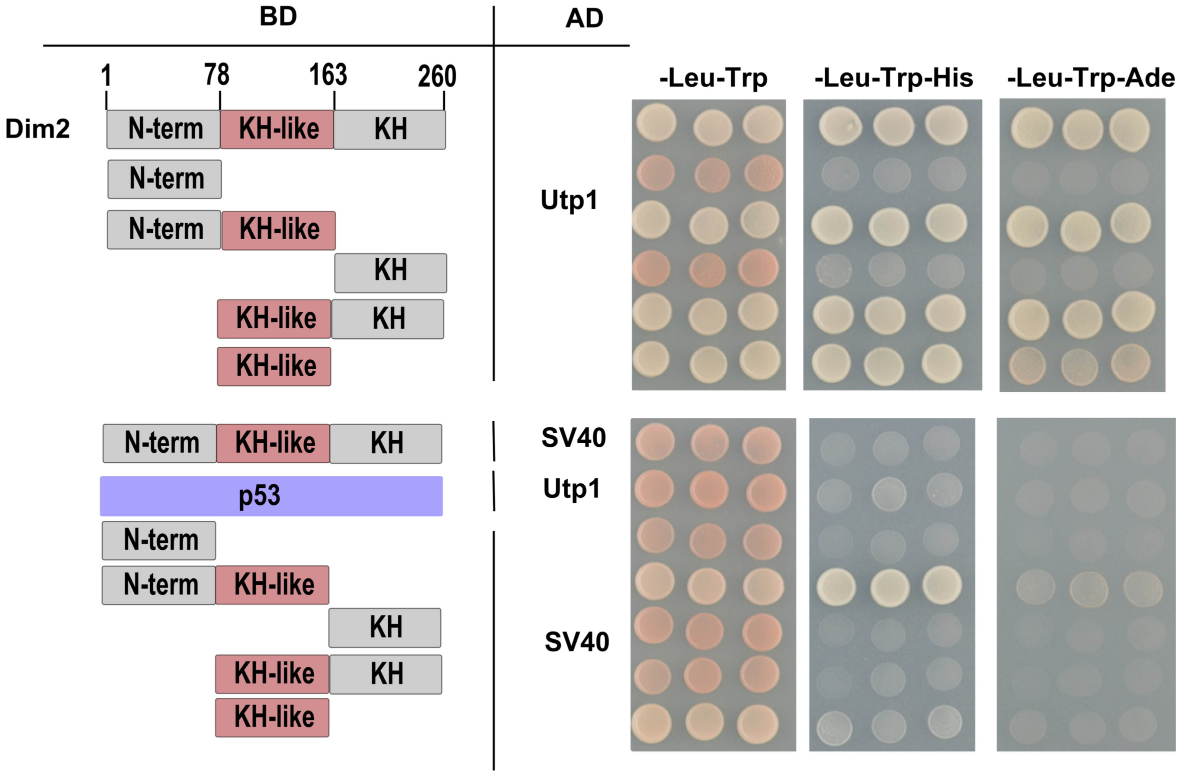

Supplementary Figure 4 Dim2 utilizes its KH-like domain for interaction with Utp1. Yeast 2-hybrid tests using Dim2 and Utp1 constructs. Three individual transformants, derived from the tester strain PJ69-4a and harboring the indicated bait (BD) and prey (AD) plasmids, were analyzed by growth on SDC-Leu-Trp (plating efficiency), SDC-Leu-Trp-His (weak interaction) and SDC-Leu-Trp-Ade plates (strong interaction). SV40 and p53 served as negative controls. The growth was analyzed after two days at 30°C.

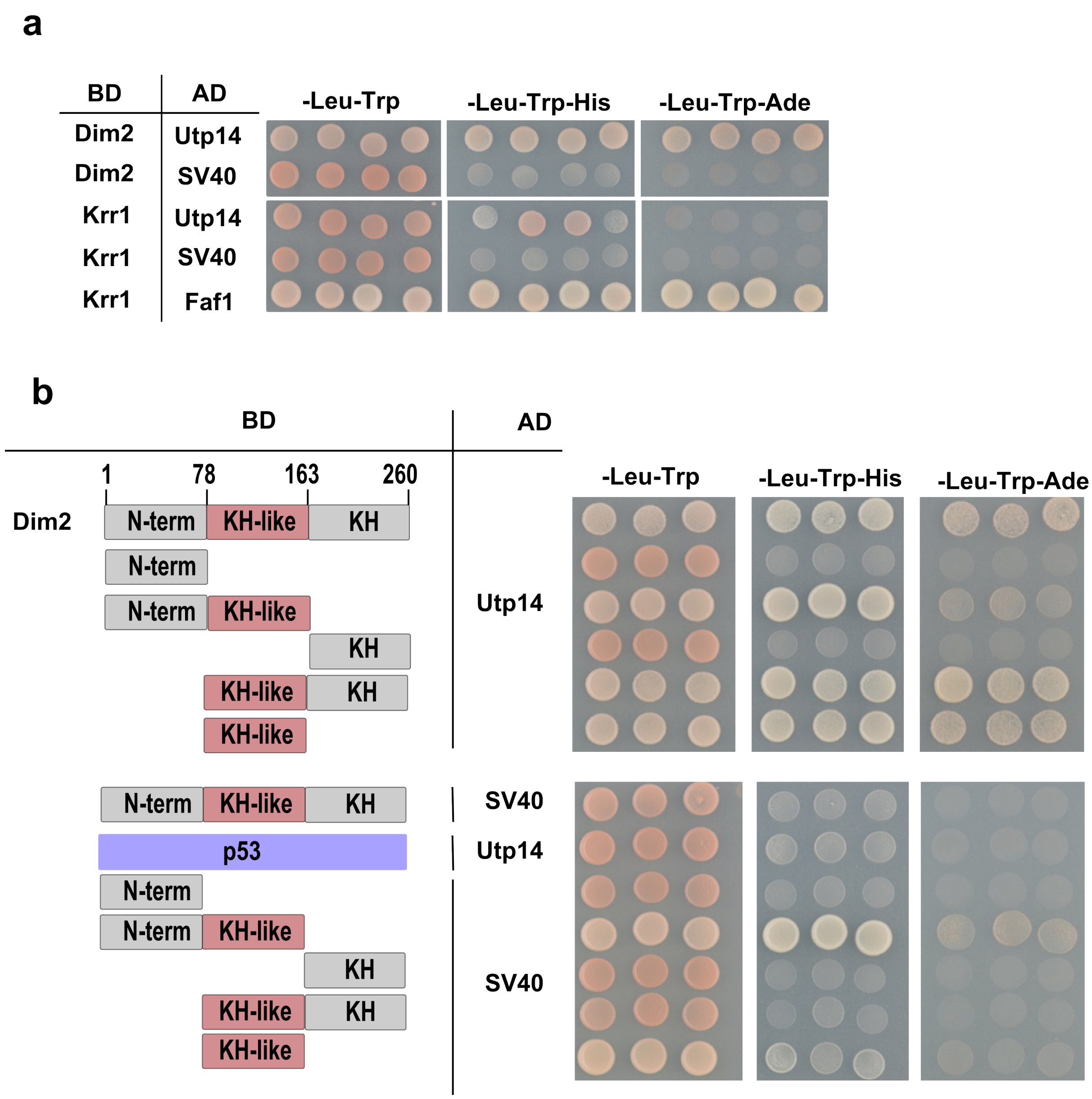

Supplementary Figure 5. Dim2 interacts via its KH-like domain with Utp14. (a, b) Yeast 2-hybrid tests using the indicated Dim2 and Utp14 constructs. Three individual transformants, derived from the tester strain PJ69-4a and harboring the indicated bait (BD) and prey (AD) plasmids, were analyzed by growth on SDC-Leu-Trp (plating efficiency), SDC-Leu-Trp-His (weak interaction) and SDC-Leu-Trp-Ade plates (strong interaction). SV40 and p53 served as negative controls. The growth was analyzed after two days at 30°C.

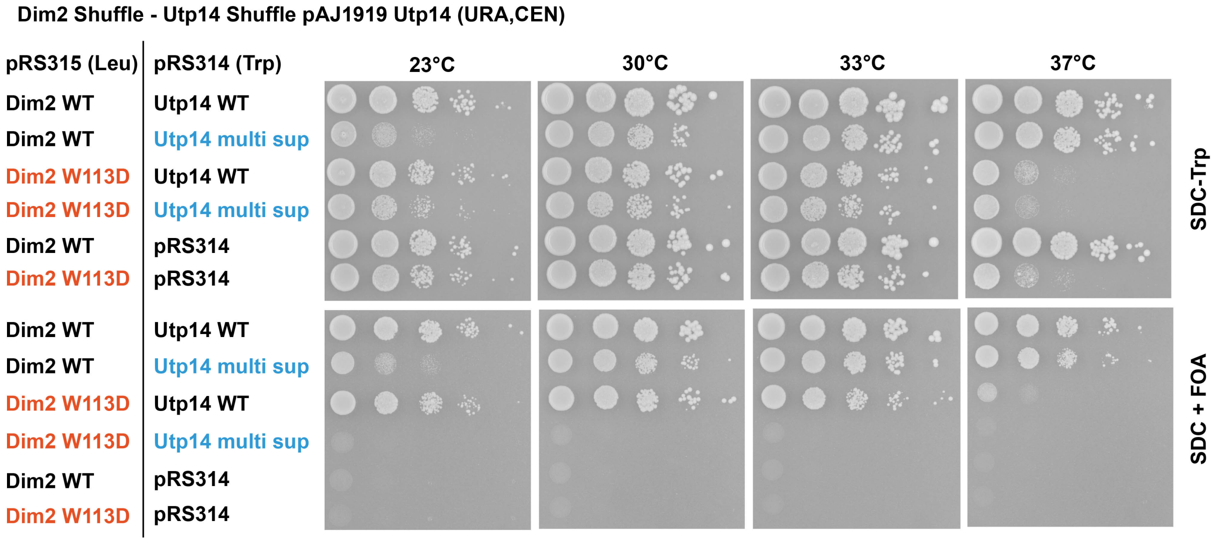

Supplementary Figure 6. Synthetic lethal interaction between mutant alleles, Dim2 W113D and Utp14 multi sup. The genetic analysis was carried out with a *dim2Δ utp14Δ* double shuffle strain, transformed with the indicated plasmids carrying *DIM2* wild-type or *dim2* W113D mutant alleles, and *UTP14* wild-type *utp14* multi sup mutant. Cells were grown on SDC-Trp or SDC + FOA plates. Growth was monitored at the indicated temperatures after 3 days. No growth on 5-FOA plates indicates synthetic lethality.

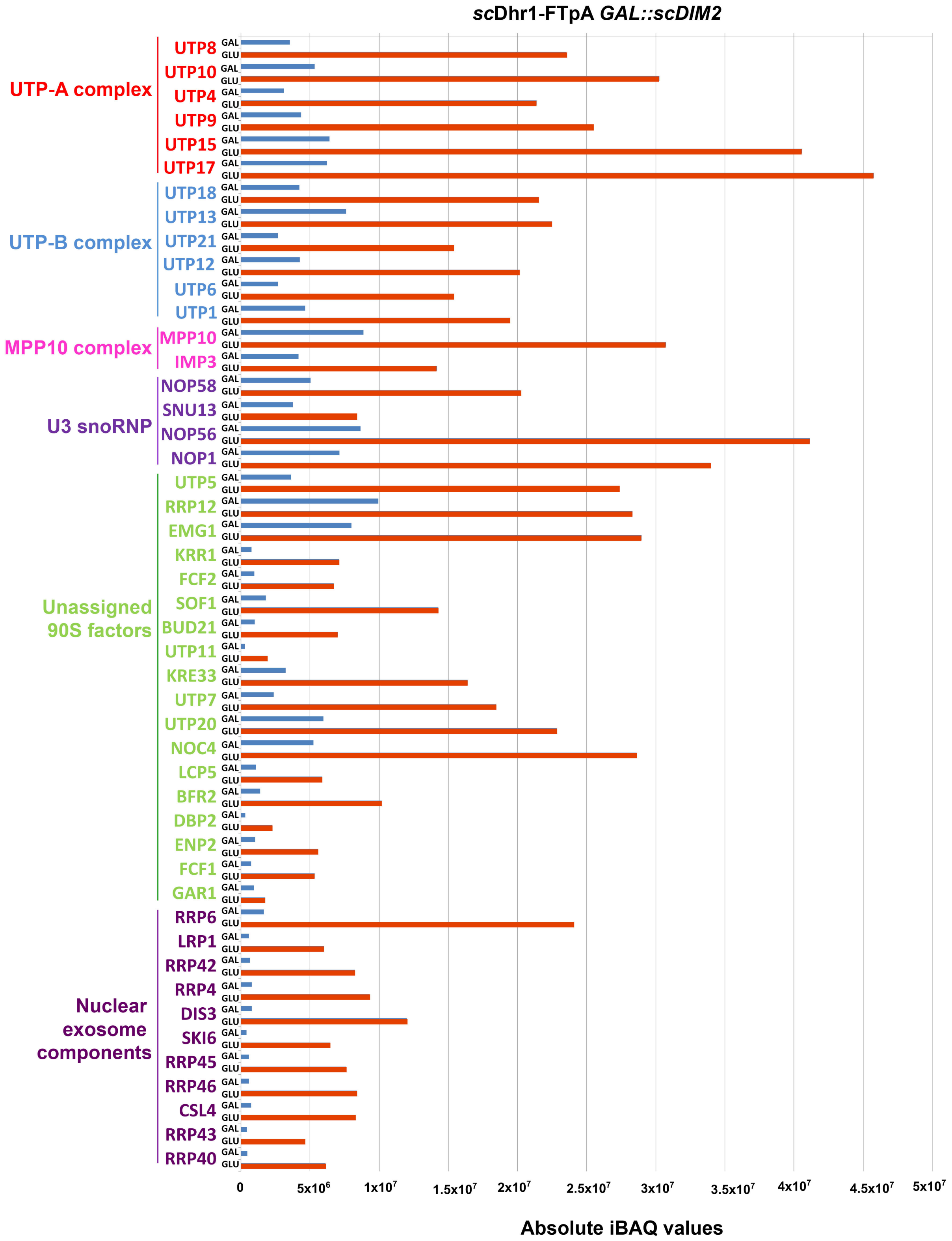

Supplementary Figure 7. Dhr1 co-purifies increased amounts of 90S factors upon Dim2 depletion. Semi-quantitative mass spectrometry of the Dhr1-FTpA eluates derived from experiment Fig. 3c. The iBAQ (Intensity Based Absolute Quantification) numbers derived from semi-quantitative mass

spectrometry analysis of the Dim2 expressing (GAL) and Dim2 depletion (GLU) conditions were normalized to the iBAQ value of the Dhr1 bait protein. The absolut iBAQ values of co-purified 90S factors derived from depleted versus expressing Dim2 cells are shown. The corresponding numerical data are shown in Supplementary Data 1.

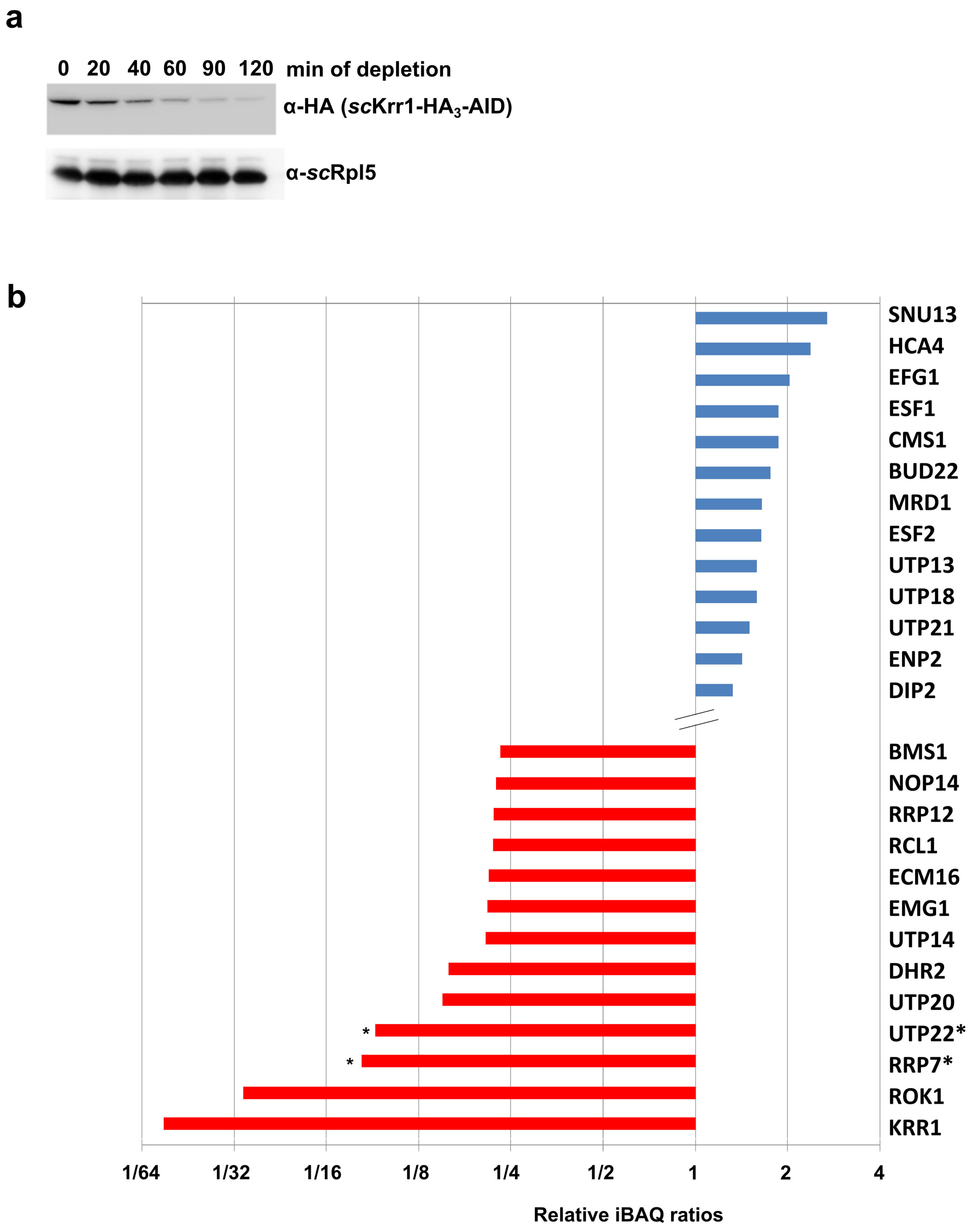

Supplementary Figure 8. Krr1 depletion leads to a reduction of UTP-C complex members Utp22 and Rrp7. (a) Western blot analysis revealing Krr1 depletion in yeast cells. *sc*Krr1-HA$_3$-AID degron strain was grown in YPD medium, before degradation of Krr1 was induced by addition of indole-3-acetic acid (Auxin) to the medium. Cells were collected at indicated time

points (min of depletion). Whole cell lysates were analyzed by SDS-PAGE and Western blotting utilizing the indicated antibodies. (b) Semi-quantitative mass spectrometry of the Utp10-FTpA eluates derived from *sc*Krr1-HA_3-AID expressing (- Auxin) and *sc*Krr1-HA_3-AID depleted (+ Auxin) cells (see Fig. 4c, lane 1 and 2). The iBAQ (Intensity Based Absolute Quantification) numbers derived from semi-quantitative mass spectrometry analysis of the two different Utp10-FTpA eluates were normalized to the iBAQ value of the Utp10 bait protein. The relative iBAQ ratio of co-purified 90S factors derived from depleted versus expressing Krr1 cells are shown. The corresponding numerical data are shown in Supplementary Data 1.

Figure 1a

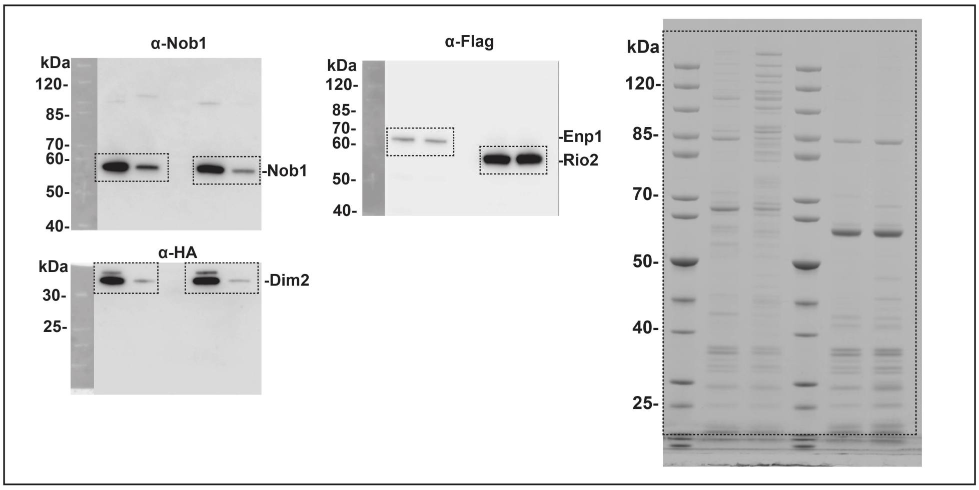

Figure 1b

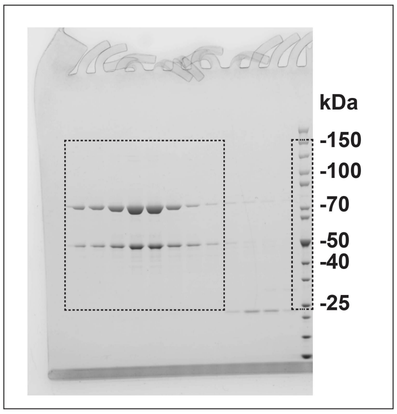

Figure 1d

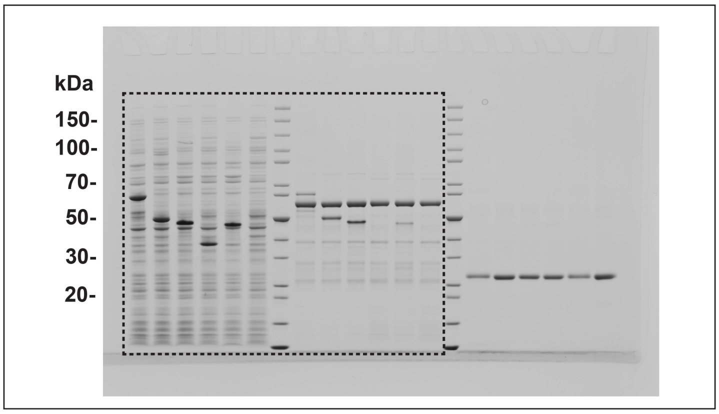

Figure 1e

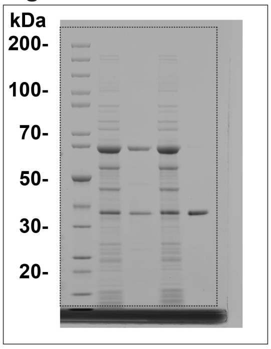

Figure 2a

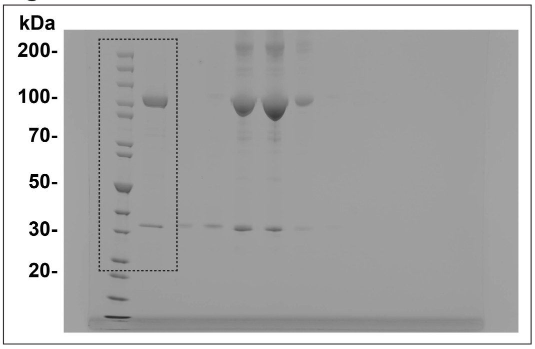

Figure 2c

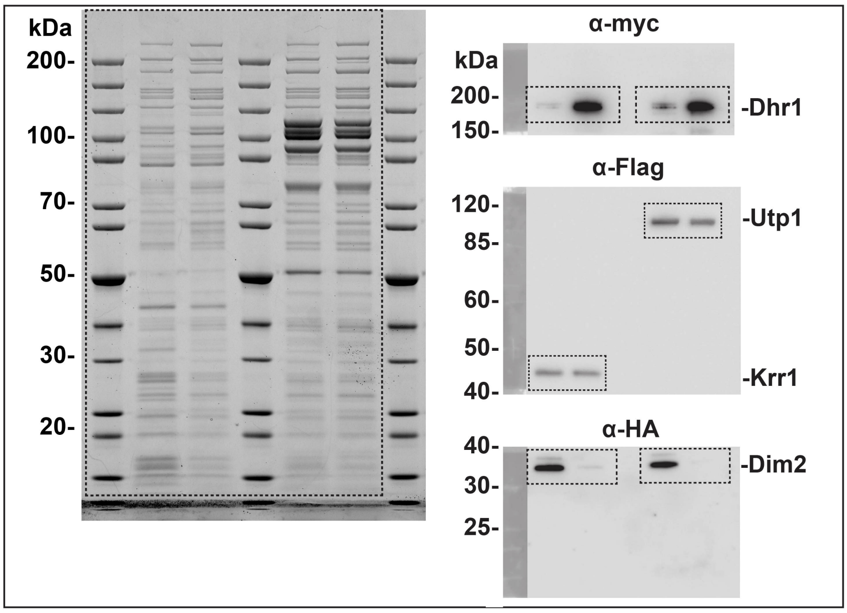

Figure 3b

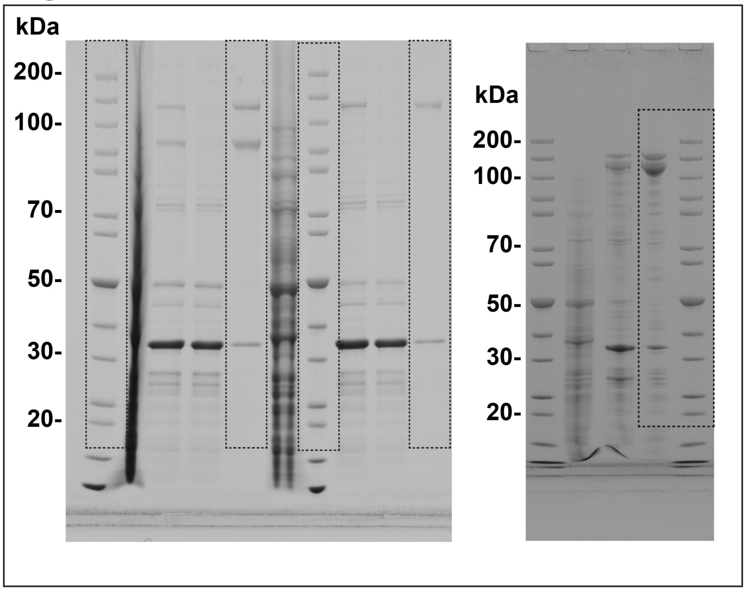

Figure 3c

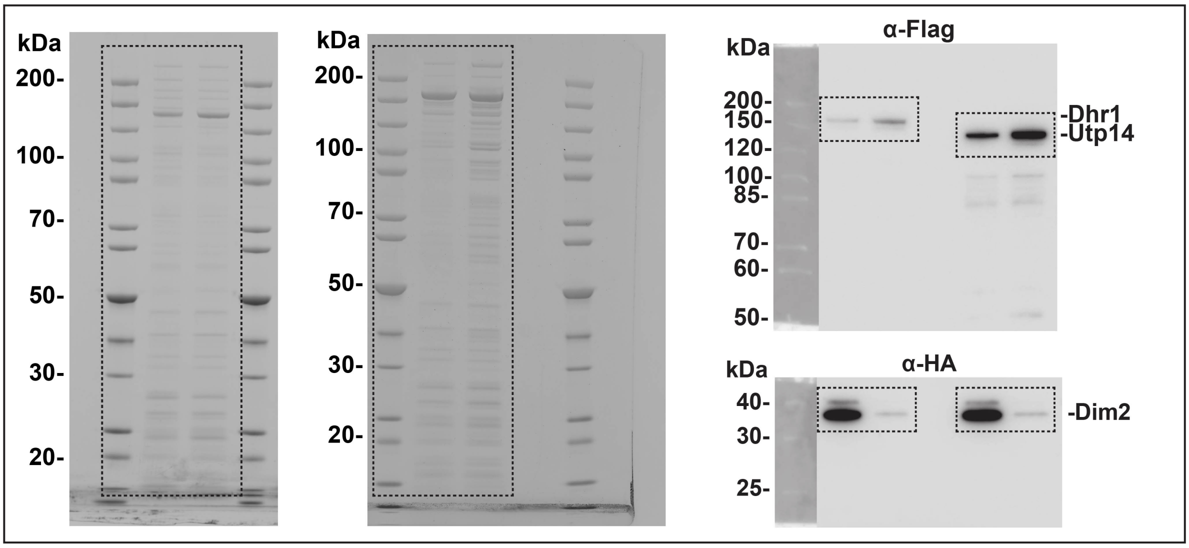

Figure 3d

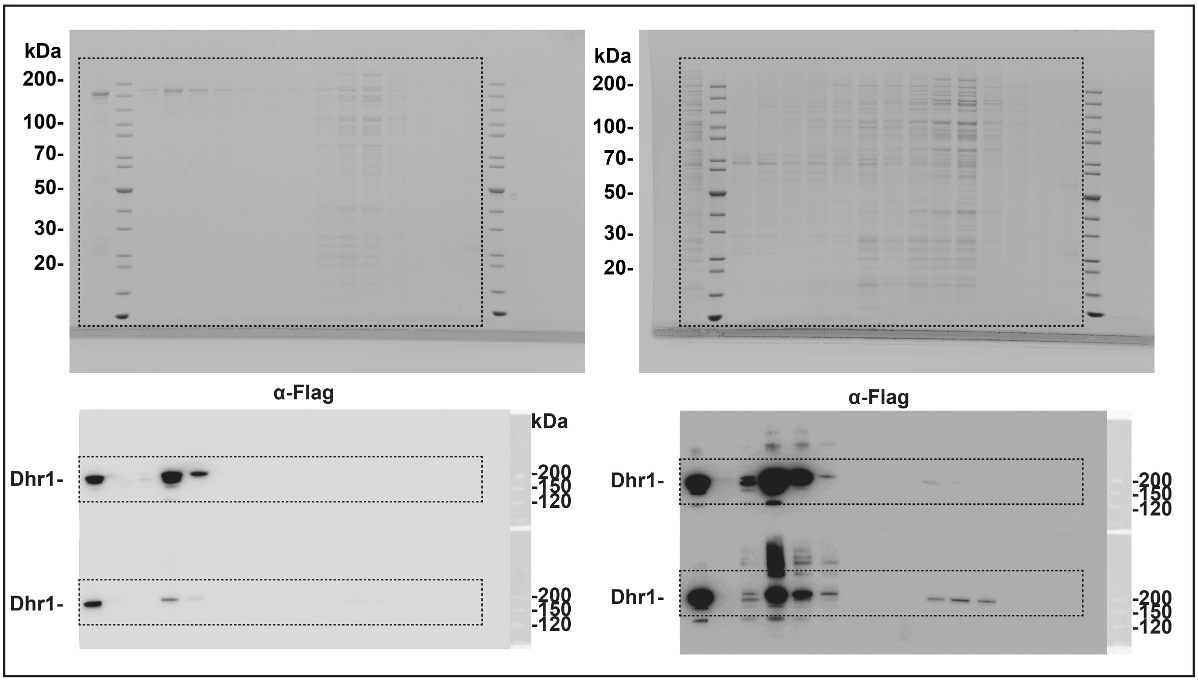

Figure 4a

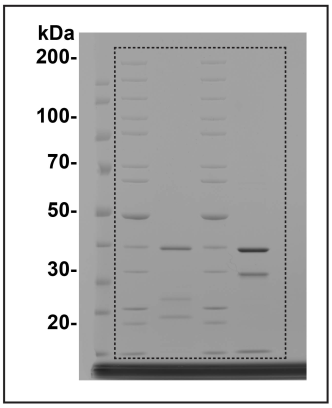

Figure 4b

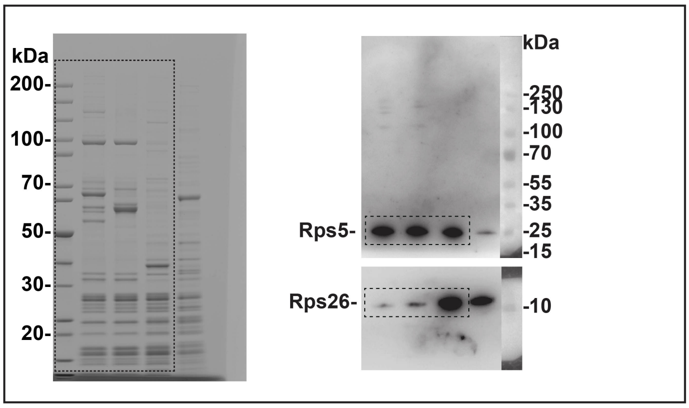

Figure 5a

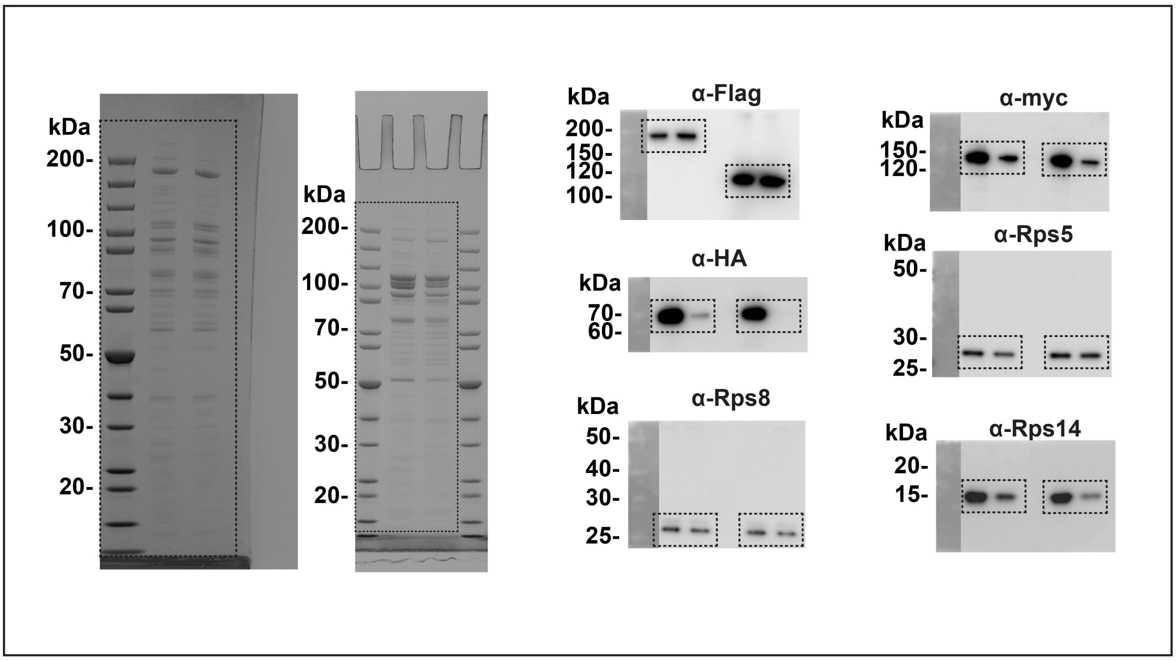

Figure 5b

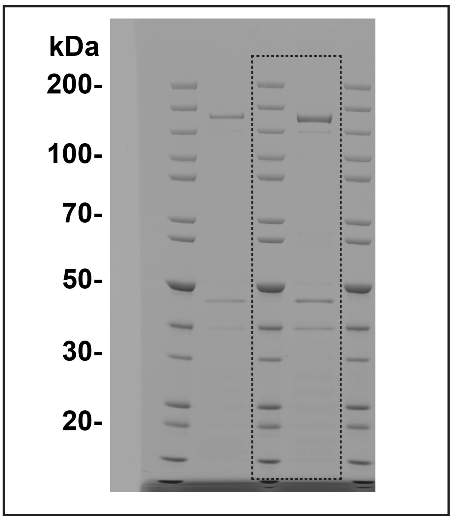

Supplementary Figure 9. Uncropped scans of Western blots and Coomassie-stained SDS-polyacrylamide gels included in main figures.

Supplementary Table 1: Plasmids used in this study

**Name**	**Genotype**	**Reference**
YCPlac111 HA-*sc*Dim2	*CEN; LEU2, AmpR, P_{GAL} HA-DIM2*	This study
petMBP-*ct*Dim2	*KanR, P_{T7} MBP-ctDIM2*	This study
petDuet HIS_6-*ct*Nob1 1-354	*AmpR, P_{T7} HIS_6-ctNOB1 1-354*	This study
pet24d *ct*Dim2	*KanR, P_{T7} ctDIM2*	This study
petDuet HIS_6-*ct*Nob1 250-354 , *ct*Nob1 1-169	*AmpR, P_{T7} HIS_6-ctNOB1 250-354, P_{T7} ctNOB1 1-169*	This study
pMT-*LEU2* ProtA-TEV-*ct*Dim2	*2µ, LEU2, AmpR, $P_{GAL1-10}$ PROTA-TEV-ctDIM2*	This study
pMT-*TRP1* $Flag_3$-*ct*Utp1	*2µ, TRP1, AmpR, $P_{GAL1-10}$ $FLAG_3$ -ctUTP1*	This study
pGBKT7 *ct*Dim2	*2µ, TRP1, KanR, GAL4BD-cMyc-ctDIM2*	Ref.[2]
pGADT7 *ct*Utp14	*2µ, LEU2, AmpR, GAL4AD-HA_3-ctDIM2*	Ref.[2]
pVA3-1	*2µ, TRP1, AmpR, GAL4 DNA-BD-MURINE p53 (72-390)*	Clontech Laboratories, Inc
pTD1-1	*2µ, LEU2, KanR, GAL4 AD-SV40 (84-708)*	Clontech Laboratories, Inc
pMT-*TRP1* $Flag_3$-*ct*Utp14	*2µ, TRP1, AmpR, $P_{GAL1-10}$ $FLAG_3$ ctUTP14*	This study
pMT-*URA3* *ct*Utp14	*2µ, URA3, AmpR, $P_{GAL1-10}$ ctUTP14*	This study
pMT-*URA3* HA_3-*ct*Dhr1	*2µ, URA3, AmpR, $P_{GAL1-10}$ HA_3-ctDHR1*	This study
pMT-*LEU2* ProtA-TEV-*ct*Krr1	*2µ, LEU2, AmpR, $P_{GAL1-10}$ PROTA-TEV-ctKRR1*	This study
pMT-*TRP1* $Flag_3$-*ct*Rps14	*2µ, TRP1, AmpR, $P_{GAL1-10}$ $FLAG_3$ ctRPS14*	This study
pMT-*URA3* *ct*Rps1	*2µ, URA3, AmpR, $P_{GAL1-10}$ ctUtp22*	This study
pMT-*TRP1* $Flag_{3x}$-*ct*Fap7	*2µ, TRP1, AmpR, $P_{GAL1-10}$ $FLAG_3$ ctFAP7*	This study
pMT-*TRP1* $Flag_3$-*ct*Rrp7	*2µ, TRP1, AmpR, $P_{GAL1-10}$ $FLAG_3$ ctRRP7*	This study
pMT-*URA3* HA_3-*ct*Utp22	*2µ, URA3, AmpR, $P_{GAL1-10}$ HA_3-ctUTP22*	This study
pGBKT7 *ct*Dim2 N-term	*2µ, TRP1, KanR, GAL4BD-cMyc-ctDIM2 1-78 aa*	This study
pGBKT7 *ct*Dim2 KHlike	*2µ, TRP1, KanR, GAL4BD-cMyc-ctDIM2 79-163*	This study

pGBKT7 *ct*Dim2 Cterm	*2µ, TRP1, KanR, GAL4BD-cMyc-ctDIM2 164-260*	This study
pGBKT7 *ct*Dim2 N + KHlike	*2µ, TRP1, KanR, GAL4BD-cMyc-ctDIM2 1-163*	This study
pGBKT7 *ct*Dim2 KHlike + Cterm	*2µ, TRP1, KanR, GAL4BD-cMyc-ctDIM2 79-260*	This study
pGBKT7 *ct*Krr1	*2µ, TRP1, KanR, GAL4BD-cMyc-ctKRR1*	Ref.[2]
pGADT7 *ct*Faf1	*2µ, LEU2, AmpR, GAL4AD-*HA_3*-ctFAF1*	Ref.[2]
pRS315	*CEN, LEU2, AmpR*	Ref.[3]
pRS316	*CEN,URA3, AmpR*	Ref.[4]
pRS315 *sc*Dim2	*CEN, LEU2, AmpR* P_{DIM2}*-DIM2*	This study
pRS316 *sc*Dim2	*CEN,URA3, AmpR* P_{DIM2}*-DIM2*	This study
pAJ1919 *sc*UTP14	*CEN, UR3, AmpR,* P_{UTP14}*-UTP14*	Ref.[5]
pRS314, *sc*Utp14	*CEN,TRP1, AmpR* P_{UTP14}*-UTP14*	This study, derived from Ref.[5]
pRS314, *sc*Utp14 multi sup	*CEN,TRP1, AmpR* P_{UTP14}*-UTP14 multi sup*	This study, derived from Ref.[5]
pRS315 *sc*Dim2 W113D	*CEN, LEU2, AmpR* P_{DIM2}*-DIM2*	This study
pRS315 *sc*Nob1	*CEN, LEU2, AmpR* P_{NOB1}*-NOB1*	Ref.[6]
pRS315 *sc*Nob1 *Δ 217-229*	*CEN, LEU2, AmpR* P_{NOB1}*-NOB1 Δ 217-229*	This study
pRS315 *sc*Nob1 *Δ 208-232*	*CEN, LEU2, AmpR* P_{NOB1}*-NOB1 Δ 208-232*	This study
pRS315 *sc*Nob1 *Δ 208-264*	*CEN, LEU2, AmpR* P_{NOB1}*-NOB1 Δ 208-264*	This study
pet24a HIS_6-*ct*Nob1 1-476	*KanR,* P_{T7} *ctNOB1 1-476*	This study
pet24a HIS_6-*ct*Nob1 169-476	*KanR,* P_{T7} *ctNOB1 169-476*	This study
pet24a HIS_6-*ct*Nob1 1-355	*KanR,* P_{T7} *ctNOB1 1-355*	This study
pet24a HIS_6-*ct*Nob1 1-294	*KanR,* P_{T7} *ctNOB1 1-294*	This study

pet24a HIS_6-*ct*Nob1 1-250	*KanR, P_{T7} ctNOB1 1-250*	This study
pet24a HIS_6-*sc*Nob1	*KanR, P_{T7} scNOB1*	This study
pet24a HIS_6-*sc*Nob1 Δ 208-264 (Δ MID)	*KanR, P_{T7} scNOB1 Δ 208-264*	This study
petDuet HIS_6.*ct*Dim2, *ct*Nob1	*AmpR, P_{T7} HIS_6-ctDIM2, P_{T7} ctNOB1*	This study
petDuet HIS_6.*ct*Dim2, *ct*Nob1 W267G	*AmpR, P_{T7} HIS_6-ctDIM2, P_{T7} ctNOB1 W267G*	This study
pGADT7 *ct*Utp1	*2µ, LEU2, AmpR, GAL4AD-HA_3-ctDIM2*	Ref.[2]
pGADT7 *ct*Dhr1	*2µ, LEU2, AmpR, GAL4AD-HA_3-ctDIM2*	Ref.[2]
pet24d GST-TEV-*ct*Dim2	*KanR, P_{T7} GST-TEV.ctDIM2*	This study
pet24d GST-TEV-*sc*Dim2	*KanR, P_{T7} GST-TEV.scDIM2*	This study

Supplementary Table 2: Yeast strains used in this study

**Name**	**Genotype**	**Reference**
W303 α	*Matα, ura3-1, trp1-1, his3-11,15, leu2-3,112, ade2-1, can1-100, GAL+*	Ref.[4]
PJ69-4a	trp1–901, leu2–3,112, ura3-52, his3-200, gal4D, gal80D, LYS2::GAL1–HIS3, GAL2–ADE2, met2::GAL7-lacZ	Ref.[7]
Enp1-FTpA, *dim2* Δ shuffle	W303, *Matα, dim2::natNT2, enp1-FTpA::HIS3, YCPlac111 HA-scDim2*	This study
Rio2-FTpA, *dim2* Δ shuffle	W303, *Matα, dim2::natNT2, rio2-FTpA::HIS3, YCPlac111 HA-scDim2*	This study
Krr1-FTpA, *dim2* Δ shuffle	W303, *Matα, dim2::natNT2, krr1-FTpA::HIS3, YCPlac111 HA-scDim2*	This study
Utp1-FTpA, *dim2* Δ shuffle	W303, *Matα, dim2::natNT2, utp1-FTpA::HIS3, YCPlac111 HA-scDim2*	This study
Dhr1-FTpA, *dim2* Δ shuffle	W303, *Matα, dim2::natNT2, dhr1-FTpA::HIS3, YCPlac111 HA-scDim2*	This study
Utp14-FTpA, *dim2* Δ shuffle	W303, *Matα, dim2::natNT2, utp14-FTpA::HIS3, YCPlac111 HA-scDim2*	This study
Asc1-FTpA	W303, *Matα, Asc1-FTpA::HIS3,*	This study

Utp10-FTpA, *Krr1*-HA_3-AID, *Utp22*-myc_{13}	W303, *Matα, Utp10-FTpA::natNT2, , P_{ADH1}-OSTIR1-9xmyc::TRP1, Krr1-HA_3-AID::HIS3MX6, Utp22-13MYC-kanMX6*	This study
Krr1-FTpA, *dim2* Δ shuffle, Dhr1-myc_{13}	W303, *Matα, dim2::natNT2, krr1-FTpA::HIS3, Dhr1-13MYC-kanMX6, YCPlac111 HA-scDim2*	This study
Utp1-FTpA, *dim2* Δ shuffle , Dhr1-myc_{13}	W303, *Matα, dim2::natNT2, utp1-FTpA::HIS3, Dhr1-13MYC-kanMX6, YCPlac111 HA-scDim2*	This study
dim2 Δ *utp14* Δ double shuffle, pRS315 scDim2	W303, *Matα, dim2::natNT2, utp14:: HIS3, pRS315 scDim2, pAJ1919 scUTP14*	This study
dim2 Δ *utp14* Δ double shuffle, pRS315 scDim2 W113D	W303, *Matα, dim2::natNT2, utp14:: HIS3, pRS315 scDim2 W113D, pAJ1919 scUTP14*	This study
nob1 Δ shuffle	W303, *Mata, nob1::kanMX4, pRS316 scNob1*	Ref.[6]
Ltv1-FTpA, *nob1* Δ shuffle	W303, *Mata, nob1::kanMX4, pRS316 scNob1, FTpA::HIS3*	This study
Utp1-FTpA, *Krr1*-HA_3-AID, *Utp22*-myc_{13}	W303, *Matα, Utp10-FTpA::natNT2, , P_{ADH1}-OSTIR1-9xmyc::TRP1, Krr1-HA_3-AID::HIS3MX6, Utp22-13MYC-kanMX6*	This study
Enp1-FTpA, *dim2* Δ shuffle	W303, *Matα, dim2::natNT2, enp1-FTpA::HIS3, pRS316 scDim2*	This study
Rio2-FTpA, *dim2* Δ shuffle	W303, *Matα, dim2::natNT2, rio2-FTpA::HIS3, pRS316 scDim2*	This study

Supplementary - References

1. Waterhouse, A.M., Procter, J.B., Martin, D.M., Clamp, M. & Barton, G.J. Jalview Version 2--a multiple sequence alignment editor and analysis workbench. *Bioinformatics* **25**, 1189-91 (2009).
2. Bassler, J. et al. Interaction network of the ribosome assembly machinery from a eukaryotic thermophile. *Protein Sci* **26**, 327-342 (2017).
3. Sikorski, R.S. & Hieter, R. A system of shuttle vectors and yeast host strains designed for efficient manipulation of DNA in Saccharomyces cerevisiae. *Genetics* **122**, 19-27 (1989).
4. Thomas, B.J. & Rothstein, R. Elevated recombination rates in transcriptionally active DNA. *Cell* **56**, 619-30 (1989).
5. Zhu, J., Liu, X., Anjos, M., Correll, C.C. & Johnson, A.W. Utp14 Recruits and Activates the RNA Helicase Dhr1 To Undock U3 snoRNA from the Preribosome. *Mol Cell Biol* **36**, 965-78 (2016).
6. Pertschy, B. et al. RNA helicase Prp43 and its co-factor Pfa1 promote 20S to 18S rRNA processing catalyzed by the endonuclease Nob1. *J Biol Chem* (2009).
7. James, P., Halladay, J. & Craig, E.A. Genomic libraries and a host strain designed for highly efficient two-hybrid selection in yeast. *Genetics* **144**, 1425-36 (1996).
