# Supplementary material for: Interdependent action of KH domain proteins Krr1 and Dim2 drive the 40S platform assembly
Source: Nat Commun. 2017 Dec 20;8:2213. doi: 10.1038/s41467-017-02199-4 (PMC5738357; doi:10.1038/s41467-017-02199-4)
Supplement: Supplementary file 2 — Description of Additional Supplementary Files [file 41467_2017_2199_MOESM2_ESM.pdf]

Descriptions of Additional Supplementary Files

File Name: Supplementary Dataset 1
Description: Supplementary Data 1 contains the complete lists of proteins and their respective iBAQ values found in semi-quantitative mass spectrometry analysis underlying Figure 2d, Supplementary Figure 7 and Supplementary Figure 8b.
